# The construction of Chinese microblog gender-specific thesauruses and user gender classification

**DOI:** 10.1007/s41109-018-0104-1

**Published:** 2018-11-08

**Authors:** Zhiliang Zhu, Zejun Ke, Jiayin Cui, Hai Yu, Guoqi Liu

**Affiliations:** Software College, Northeastern University, Shenyang, China

**Keywords:** Gender classification, Statistical feature, Gender-specific thesaurus, Machine learning

## Abstract

Based on the statistical features, short text messages published by different gender users are different in terms of the words and semantics used. In this paper, two new features are constructed after constructing a gender-specific thesaurus. A new classification model is constructed by combining the traditional statistical features and the improved text implicitness feature. The experimental evaluation performed on the Sina Weibo dataset demonstrated the effectiveness of gender-specific thesaurus-based features, and the improved text implicitness feature improved the accuracy of gender classification to 84.7%.

## Introduction

With the popularization and rapid development of the Internet, social networks are favored and sought after by many Internet users due to their unique virtuality, diversity, innovation, freedom and alienation. Foreign social networks are represented by platforms such as Facebook, Twitter and Instagram, while domestic ones are represented by Sina Weibo, Tencent Weibo, Wechat, Baidu Post Bar and Zhihu. In particular, anonymity is an important feature of social networks. People may not need to provide their real identities in cyberspace, such as their names, ages, genders, and addresses. However, while social networks are growing, the drawbacks of anonymous remarks are constantly being magnified and exploited. Users are vulnerable to anonymous and fraudulent attacks when socializing online, including receiving false information and even being mentally or physically challenged. In many criminal cases, the perpetrators attempt to hide their addresses by using anonymous servers that hide their true identity. Therefore, it is imperative to design an effective identity tracking method for cyberspace forensics. One of the most important aspects of this is gender classification.

In addition to the value of Internet user security, gender classification of users in social networks is also crucial to market intelligence. User’s gender information can be used in targeted advertising and product development, thereby improving the accuracy of personalized recommendations and enabling more effective business promotion and accurate ad serving. In scientific research, this information can provide the foundation for the separation of gender topics, the discovery of gender hot words, behavioral analysis and emotional analysis.

Currently, scholars usually construct features by using statistical analysis methods and semantic analysis methods. There is not much research on gender classification in Chinese because Chinese is much more complex than English. Furthermore, Chinese people are used to being euphemistic when they express themselves. In terms of resources, the Chinese public thesaurus is relatively limited. Therefore, gender classification research in Chinese is quite difficult. Based on the Sina Weibo dataset, we build a gender-specific thesaurus to provide resources for scholars to perform gender classification research in the future. Moreover, this paper allows the more accurate calculation of the implicitness in the Chinese language by improving the text implicitness calculation. Then, we combine some traditional statistics-based text features and expression features to construct the feature vectors for gender classification.

Of course, our research focuses on normal gender recognition without regard to gender camouflage (i.e., one gender deliberately presents another person’s characteristics) because we are based on user characteristics for gender recognition, if a person disguise the features provided, then we are not extracting the correct features, naturally can not correctly identify the user’s gender, but this kind of gender camouflage is only a small part, so this article does not consider this complicated situation.

This paper is organized as follows. “Related work” section surveys existing work in gender classification. “The extraction of a gender-specific thesaurus and the construction of feature vectors” section presents the construction of the feature matrix. “Experimental process and result analysis” section describes the model that we build, presents our experimental results, and analyzes the experiment. Finally, “Conclusions” section summarizes our findings and conclusions.

## Related work

In recent years, although research on gender classification based on social networks has not been popular, related works have made some progress. Because of the differences in language, Before 2018, the Chinese word segmentation was word-level. In 2018, some scholars tried to reach the character-like level (Cao 2018), but Chinese only had semantic meaning above a single text level. If it was divided further, the original meaning was destroyed. Moreover, for Chinese language expression, the basic unit is more of a word level, and most language analyses except Chinese can reach the character level. Therefore, Chinese gender classification based on NLP is different from other languages.

In the research of Chinese gender classifications, Liu and Niu (2016) proposed a gender identification method based on the feature extraction of emotional words and emotion-related language style. Huang et al. (2014) proposed a microblog message representation model based on a tolerance rough set, constructed a feature vector by extracting gender-based feature differences in rough sets, and finally used the k-NN classifier to classify the experiments. Compared with the characteristic term frequency representation model, the accuracy rate is 7%. Tang and Lin (2010) achieved gender recognition based on different descriptions of men or women in various aspects. Qi (2017) selected the corpus of Tencent Weibo to extract the vocabulary dependency of short texts and compared it with the vocabulary features of the existing documents to some extent. This avoided the sparsity of short text feature sets, and the use of machine learning (such as the SVM Algorithm) was experimentally verified. Song et al. (2016) constructed the LDA model and trained user content, attention and interest topics for gender identification. Yao (2017) used four classification algorithms to identify the microblog users for gender recognition through the selection of feature words and TF-IDF scores, thus achieving a highest classification accuracy of 79%.

In the field of gender recognition in English, some foreign scholars have achieved limited results. Aravantinou et al. (2015) conducted large-scale and fine-grained statistical analysis on a corpus, constructed feature vectors at the character level, applied the POS annotation category and the N-Gram phrase category, and used a machine learning algorithm for feature selection and result verification. The results show that the features constructed by the N-Gram language model have higher scores among all the statistical features and that the SVM classifier performs better than other classifiers. Burger et al. (2011) use the content of the tweet text and the three fields in the Twitter user profile: full name, screen name and description as features to classify Twitter users. Schwartz et al. (2013) use differential language analysis (DLA), to find language features. They extract 700 million instances of words, phrases, and automatically generated topics and correlate them with gender, age, and personality. They present a word cloud-based technique to visualize results of DLA. Montero et al. (2014) incorporated the feature attributes based on emotions, used the SVM classifier to test the results and obtained a recognition accuracy of 80%. Mukherjee and Liu. (2010) introduced the text implicitness feature proposed by Heylighen and Dewaele (2002) based on the use of traditional semantic statistics to construct feature vectors and proposed a new method to find POS sequences with strong constraints as new features. They improved the recognition accuracy of the algorithm to reach a highest classification accuracy of 88.56%. There are the competition about identifying age, gender, and personality traits of Twitter users. Rangel (2015) overview the framework and the results for the Author Profiling Shared Task organised at PAN 2015. They presents the approaches of 22 participants. Bamman et al. (2014) present a study of the relationship between gender, language, and social network connections in social media text. They use a novel corpus of more than 14,000 individuals on Twitter, and perform a computational analysis of the impact of gender on both their lexical choices and their social networks. They address two limitations of previous quantitative analyses of language and gender.

Although many scholars have explored the field of gender recognition in Chinese, there are still some limitations. And so far, Chinese related dictionary material is still lacking, so the focus of this article is on the construction of gender-specific thesaurus and then classify users by machine learning based on the built dictionary and related features extracted from Weibo. To achieve the goal of gender identification.

## The extraction of a gender-specific thesaurus and the construction of feature vectors

In previous studies, some scholars deliberately deleted some seemingly ordinary features in order to reduce the impact of noise on the model, increase the accuracy of the model, and reduce the training time. With the development of big data, increasing numbers of studies have found that there are interrelated relationships between features. Furthermore, the rapid development of hardware has brought support for machine learning algorithms, allowing some algorithms that were not suitable for training with huge feature vectors in the past to fulfill their potential. Additionally, based on the feature selection algorithm or the relational analysis, feature selection scores can be calculated for all the selected features based on a classifier, and feature selection can be performed according to the calculation results to improve the classification accuracy.

Based on the differences in the degrees of use of the different words in male and female data sets, this paper constructs a gender-specific thesaurus. Then, the text implicitness formula F-Measure is improved, the traditional statistical features are fused, and a 15-dimensional feature vector is constructed.

### The extraction of gender-specific thesaurus

The information in social networks is not limited to text information. With the development of social media, audio, pictures and video have also become tools for people to transmit information. However, text is still a form of data that people are accustomed to using. Therefore, information brought by text data is still of great value. In the field of gender identification, scholars also often use text data for analysis, especially semantic analysis methods, and constructing a thesaurus is an important step in semantic analysis. Currently, there are several problems in the Chinese thesaurus regarding gender identification.

(i) There are few Chinese thesauruses for gender identification. Most of these thesauruses are constructed based on a topic or emotional features and are used for sentiment analysis. Thesaurus resources are extremely scarce in gender identification;
(ii) The construction of a thesaurus is based on semi-manual annotations and the refinement of key words. However, since the official Sina Weibo beta in late 2009, people have relied on Twitter-like networks to publish increasingly more subjective comments from the media platform. Furthermore, the freedom and relaxation of the network are subtly influencing people’s originally strict and normative expressions. A wide range of online vocabulary and online styles arose. Hence, the previous method of extracting a thesaurus and the validity of this thesaurus can no longer adapt to the current gender identification research;
(iii) The existing thesauruses are mostly from the perspective of words. These thesauruses are built based on the semantic similarities between words or the similarity of emotional expressions. However, in gender identification, we mainly focus on the different words used by male and female users. Therefore, the existing thesaurus resources do not satisfy the research on gender identification.

In response to the above questions, we believe that it is necessary to construct a gender-specific thesaurus for gender identification. The construction of a thesaurus for gender identification is mainly aimed at reflecting the different wordings between male users and female users when they are expressed through the gender-specific thesaurus. Men and women, in many contexts, will show considerable differences. Thus, this paper illustrates three language scenarios.

(i) Daily concern topics
  This scenario is relatively easy to understand. Men may be more sensitive to technology, sports, state affairs, the economy, games and more. Therefore, the use of terms like “ (NBA)”, “ (policy)” and some game-specific online languages in men’s microblogging data is significantly higher than a female user’s degree of use. Since female users may be more interested in beauty and fashion information, words such as “ (lipstick)”, “ (facial mask)” and “ (shopping)” appear more frequently than male users;
(ii) The way to describe strong emotions
  In this scenario, there are two differences between men and women. First, in the use of language for emotional expression, the average frequency of female users using emotional and sad emotional words is significantly higher than that of male users, while the average frequency of male users using angry and bad mood words is obviously higher than female users (Liu and Mihalcea 2007). Second, in expressing the same emotions, the emotional words used by men and women are different. For example, with angry emotions, men generally express emotions directly, with some straightforward words, such as some dirty words, and women will use “ (silent)”, “ (want to cry)” and other similar words;
(iii) The way to describe a specific object
  There is also a marked difference in the way men and women express themselves. For example, “ (dear)” may be used when users need to address their loved one. However, “ (wife)” and “ (girlfriend)” are more often used by male users, while “ (husband)” and “ (boyfriend)” will be more used by female users.

We briefly addressed the differences between the words used by men and women in the above three scenarios. It can be seen that the thesaurus used for gender identification is able to well reflect the differences between the words of male users and female users. Nonetheless, many times, male users and female users will use the same words. Take the following four weibo as an example:

*Weibo*_11_= “! ! ! []! ! ”

Translation: I am really angry!! Oh my God! [emotion-angry] !!

*Weibo*_12_= “NBA ∘ Curry MVP”

Translation: The NBA basketball game is so exciting. Curry MVP

*Weibo*_21_= “,  ***⋯***”

Translation: You have to make me angry, really speechless...

*Weibo*_22_= “, , ”

Translation: Today, I went out and made up my makeup. My husband praised me so beautiful, oh

Through the description of the above scenario, it can be clearly seen that *Weibo*_11_ and *Weibo*_12_ is attributed to male users, *Weibo*_21_ and *Weibo*_22_ is attributed to female users. However, both and are described as angry emotions. The difference is not obvious, and we cannot judge just from the word itself whether it is from a male or female user. In addition to the semantic meanings of words, the differences in the uses of words are more often reflected in the frequencies of use of different genders. Therefore, this paper proposes to discover the exclusive vocabularies of men and women through the word frequency statistics of a large number of users. However, as the data continues to become more abundant, words used only by men or women will gradually decrease. Therefore, we can determine the ownership of words according to the degree to which words are used in the male or female corpus in order to construct a gender-specific corpus. The following will introduce the main steps in this paper to build a gender-specific thesaurus:

(i) Weibo data is stored in the corpus *WeiboDataSet*_*all*_. There are *M* microblogs, and the corpus is divided into the male corpus *WordList*_*male*_ and the female corpus *WordList*_*female*_;
(ii) Word-segment all the microblog data in the corpus to obtain *W* words, and then perform deduplication to finally obtain *W*^′^ non-repetitive words;
(iii) Finally, the partitioning function *D*(*Word*_*k*_) is used to calculate the attribution properties of words. When the ratio of the degree of *Word*_*k*_ used in *WordList*_*male*_ to the degree of its use in *WordList*_*female*_ is greater than the separation coefficient *θ*, it is judged that it belongs to the male-specific thesaurus. Likewise, the ratio of the degree that *Word*_*k*_ is used in *WordList*_*female*_ to the degree of its use in *WordList*_*male*_ is greater than the separation coefficient *θ*, it is judged as belonging to the female-exclusive thesaurus.

When

1 $$\begin{array}{@{}rcl@{}}\eta_{k} = \frac{Count\_M_{k}}{Count\_F_{k}} \end{array} $$

*D*(*Word*_*k*_) is calculated as follows:

2 $$\begin{array}{@{}rcl@{}}  D\left({Word}_{k}\right) = \left\{ \begin{array}{ll} 1 & \eta_{k} > \theta\\ -1 & \frac{1}{\eta_{k}} < \theta\\ 0 & {Others} \end{array} \right., \quad (1 < \theta \le 4) \end{array} $$

**Fig. 1:**
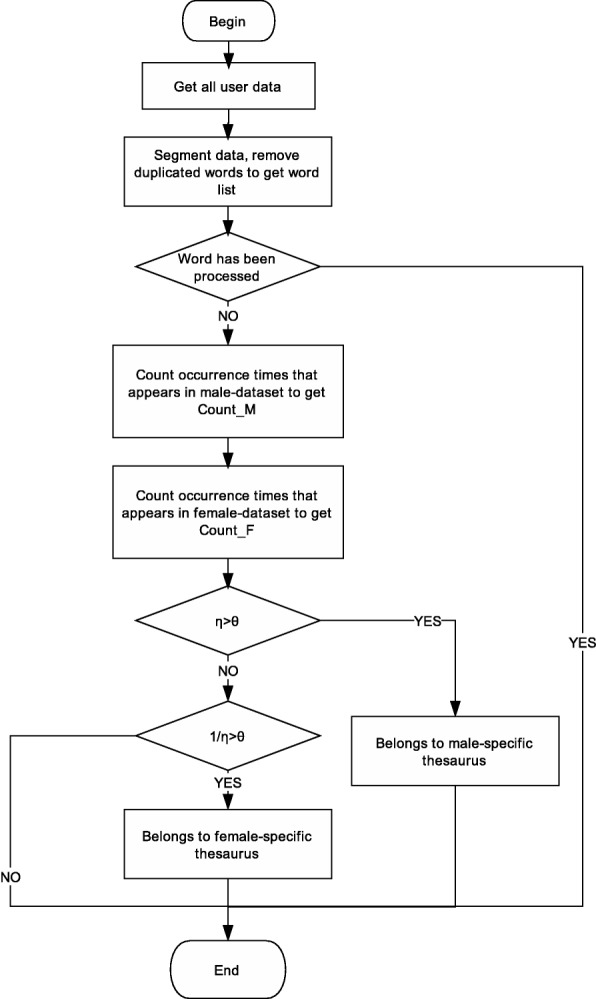
Gender-specific thesaurus construction flow chart

The flow chart of the specific construction of the gender-specific thesaurus is as shown in Fig. 1. First, all user data is processed by word segmentation, and then the repeated words are removed to obtain a list of words. Then count the number of times each word appears in male and female data. At last, count the *D*(*Word*_*k*_) of each word.

If *D*(*Word*_*k*_)=1, *Word*_*k*_ is considered a male-specific word. If *D*(*Word*_*k*_)=−1, *Word*_*k*_ is considered a female-specific word. Otherwise, *Word*_*k*_ is useless. After the above steps, different male-specific thesaurus *Exclusive*_*M* and female-specific thesaurus *Exclusive*_*F* are obtained under different *θ*.

**Fig. 2:**
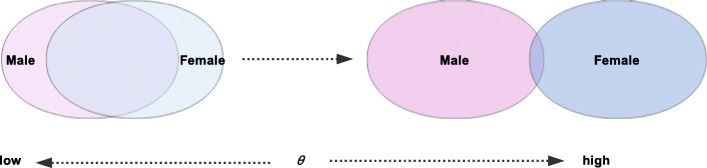
Thesaurus separation diagram

A separation factor *θ* is a value used to distinguish the degree of *Word*_*k*_’s use by men and women. The use of different separation factors *θ* can obtain different gender-specific thesauruses. As shown in Fig. 2, the purpose of using different *θ*s for the thesaurus division is to separate the gender-specific thesaurus that is originally mixed with low gender representation into a low-representative and highly representative gender-specific thesaurus.

When *θ*=1.1 and *θ*=2.0, the top-10 words in the gender-specific thesauruses are as follows in Table 1:

**Table 1:** Top-10 words in gender-specific thesauruses

*θ*	Male	Female	*θ*	Male	Female
1.1			2.0		
					
					
					
					
	Cry				
					
					
					
					

When *θ* changes dynamically, the gender-specific thesaurus changes significantly. On the one hand, when the corpus data is continuously enriched, the words contained in *WordList*_*male*_ and *WordList*_*female*_ will be continuously enriched. On the other hand, the gender-specific thesaurus obtained by using different *θ* values will be dynamically optimized. During the experiment, we set the self-growth step to 0.1 and found that the genders based on the gender-specific thesaurus had the best effects on the experimental dataset when *θ*=1.8. After constructing the gender-specific thesaurus, we can construct new features based on it and then merge some other features to perform the gender identification of the Weibo users. The elaboration and extraction of specific features are explained below.

### Feature construction based on gender-specific thesauruses

After the construction of the gender-specific thesaurus, two new features are constructed based on the frequency and universality of the use of words in the gender-specific thesaurus. The frequency refers to how many words in the user’s word set exist in the gender-specific thesaurus. Universality refers to how many distinct words in the user’s word set exist in the gender-specific thesaurus. A total of W words were obtained after segmenting user *i*’s dataset, wherein *EWM* is the number of all the words that exist in the male-specific thesaurus, and *Distinct*_*EWF* is the number of distinct words that exist in the male-specific thesaurus. If we perform the same operation based on the female-specific thesaurus, we can gain *EWF* and *Distinct*_*EWF*.

The formula of the male-specific word frequency ratio is as follows:

3 $$\begin{array}{@{}rcl@{}}  {FR}_{m} = \frac{EWM + 1}{EWM + EWF + 1} \end{array} $$

The formula of the male-specific word universality ratio is as follows:

4 $$\begin{array}{@{}rcl@{}}  {UR}_{m} = \frac{Distinct\_EWM + 1}{Distinct\_EWM + Distinct\_EWF + 1} \end{array} $$

Therefore, the improved text implicitness feature of user *i* is shown in Table 2.

**Table 2:** Gender-specific thesaurus-based feature

No.	Feature	Description
1	*F* *R*_*m*_	Male-specific word frequency ratio
2	*U* *R*_*m*_	Male-specific word universality ratio

### Improved text implicitness feature

The concept of the semantic implicitness of the text was proposed by Heylighen and Dewaele (2002) and was well applied by Nowson et al. (2005) in 2005. The method of evaluating the textual implicitness is abbreviated as the F-Measure. This method has obtained good feedback in its English context. The F-Measure is a comprehensive measure of recall and accuracy in information retrieval, and the F-Measure seeks to measure the subtlety of a piece of text based on the POS-Tag. The F-Measure’s formula is as follows:

5 $$\begin{array}{@{}rcl@{}}  F = 0.5 * [(freq.noun+freq.adj+freq.prep+freq.art)\\  - (freq.pron+freq.verb+freq.adv+freq.int) +100] \end{array} $$

where *freq.noun* indicates the number of nouns in a microblog, *freq.adj* indicates the number of adjectives in a microblog, *freq.prep* indicates the number of nouns in a microblog, *freq.prep* indicates the number of preposition in a microblog, *freq.art* denotes the number of articles in a microblog, *freq.pron* denotes the occurrences of pronouns in a microblog, *freq.verb* denotes the occurrences of verbs in a microblog, *freq.adv* indicates the occurrences of adverbs in a microblog, and *freq.int* represents the number of interjections in a microblog. According to the original formula, the appearances of pronouns, verbs, adverbs and interjections in the text will decrease the score of the F-Measure. This means that if the above four kinds of words are used excessively in one text, the context of the text will be obscured. However, if the adjectives, prepositions, nouns and articles are used more often, the user’s emotional inclination in the textual expression is more direct. When *Δf*=*F*−*σ*, a greater value of *Δf* indicates a lower degree of implicitness, which indicates that the expression of the text is more direct. The formula shows that when there are no words in a text, the value of *F* is 50, and thus *σ*=50. The women’s overall F-Measure score is lower than that of men in the large data set, indicating that women are more circumstantial and men tend to be more direct.

Inconsistent with the English context, there is no article in Chinese semantics. Therefore, if we directly use the F-Measure in Chinese, there will be a low score, and we will not be able to accurately measure the subtlety of a piece of text. To balance this defect, we incorporate the total number of emoticons into the formula to enhance the F-Measure performance in the Chinese context. In our opinion, when using emoticons, users can more directly reflect the emotions that the user wants to express (either positive or negative), and the manifestations in this context are obvious. Therefore, the use of emoticon features to fill the gap of the article is reasonable. The improved F-Measure’s formula is as follows:

6 $$\begin{array}{@{}rcl@{}}  F = 0.5 * [(freq.noun+freq.adj+freq.prep+freq.emotion) - \\ (freq.pron+freq.verb+freq.adv+freq.int) +100] - \mu \end{array} $$

To facilitate the evaluation of implicitness, let *σ*=0 and *μ*=50. There are *M* microblogs of user *i*. To calculate the F-Measure of each microblog, the F-Measure of microblog is *F*_*ij*_. Hence, the improved text implicitness *Implict* of user *i* is calculated as follows:

7 $$\begin{array}{@{}rcl@{}}  Implict = \frac{{\sum\nolimits}_{j=1}^{M} F_{ij}}{M} \end{array} $$

A greater value of results in a lower degree of implicitness. Therefore, the improved text implicitness feature of user *i* is shown in Table 3.

**Table 3:** Improved text implicitness feature

No.	Feature	Description
3	*Implict*	The text implicitness of the user

### Traditional feature

In addition to the features based on the construction of the gender-specific thesaurus and the improved semantic and text implicitness features proposed in this paper, we also need to incorporate some traditional statistical features to construct the feature vectors. The traditional statistical features are mainly based on POS tagging (Part of Speech Tagging) and semantic statistics, including statistics of nouns, prepositions and pronouns. There are a total of *N* users. Each user has *M* microblogs. The weibo *j* of user *i* gets a total of *W*_*ij*_ words after word segmentation, where the number of English words is *English*_*ij*_, the number of nouns is *Noun*_*ij*_, the number of adjectives is *Adj*_*ij*_, the number of adverbs is *Adv*_*ij*_, the number of prepositions is *Prep*_*ij*_, the number of pronouns is *Pron*_*ij*_, the number of verbs is *Verb*_*ij*_, the number of idioms is *Idiom*_*ij*_ and the number of symbols is *Sign*_*ij*_. The average number of English words for user *i* is represented as follows:

8 $$\begin{array}{@{}rcl@{}}  {English}_{avg} = \frac{{\sum\nolimits}_{j=1}^{M} {English}_{ij}}{M} \end{array} $$

Similarly, we can get the average number of nouns *Nouns*_*avg*_, the average number of adjectives *Adj*_*avg*_, the average number of adverbs *Adv*_*avg*_, the average number of prepositions *Prep*_*avg*_, the average number of pronouns *Pron*_*avg*_, the average number of verbs *Verb*_*avg*_, the average number of idioms *Idiom*_*avg*_ and the average number of symbols number *Sign*_*avg*_. The average number of words *Word*_*avg*_ of user *i* is calculated as follows:

9 $$\begin{array}{@{}rcl@{}}  {Word}_{avg} = \frac{{\sum\nolimits}_{j=1}^{M} W_{ij}}{M} \end{array} $$

The reason why Twitter-like social media platforms are popular with users is that users can post regular text messages and add information such as emoticons (client-specific emoticons, emoji emoticons, etc.), photos, audio, or video. Furthermore, emotional information, as the information interspersed in the text, is the easiest to obtain and best reflects the user’s current emotional expression from the inside. In the past, there have been gender identification research studies based on facial expressions and emotional evaluations. Studies have shown that men and women significantly differ in their choices of emoticons, emotional tones, expression categories and other aspects (Tannen 1991). We treat emoticons as a kind of “symbol” when dealing with emoticon features. That is, we only analyze statistics from the perspective of the category and attempt to discover the frequency difference between males and females when using the expression. After the microblog text is acquired, the emoticons are transformed from the “graphic emoticon” that we can see to “[text]”. By recognizing the language combination pattern of 1-4 characters embedded in “[ ]”, we can extract the number of emoticons used by the user. Hence, the emoticon frequency of user is calculated as follows:

10 $$\begin{array}{@{}rcl@{}}  {Freq}_{emoj} = \frac{{\sum\nolimits}_{j=1}^{M} {Emoj}_{ij}}{M} \end{array} $$

To summarize, the traditional features of the user *i* are shown in Table 4.

**Table 4:** Gender-specific thesaurus-based feature

No.	Feature	Description
4	*AVG*_*EWC*	The average number of English words
5	*N* *o* *u* *n*_*avg*_	The average number of nouns
6	*A* *d* *j*_*avg*_	The average number of adjectives
7	*A* *d* *v*_*avg*_	The average number of adverbs
8	*P* *r* *e* *p*_*avg*_	The average number of prepositions
9	*P* *r* *o* *n*_*avg*_	The average number of pronouns
10	*V* *e* *r* *b*_*avg*_	The average number of verbs
11	*I* *n* *t* *e* *r* *j*_*avg*_	The average number of interjections
12	*I* *d* *i* *o* *m*_*avg*_	The average number of idioms
13	*S* *i* *g* *n*_*avg*_	The average number of symbols
14	*W* *o* *r* *d*_*avg*_	The average number of words
15	*F* *r* *e* *q*_*emoj*_	The frequency of emoticons

## Experimental process and result analysis

### Data collection and model establishment

To ensure data integrity and the timeliness of information, the data set was crawled in two batches in April 2017 after modifying an open source crawler framework. First, the crawler crawls all the microblogs of the original user. After the crawling was completed, the crawler switched the target to the user’s following friends and continued the crawl. Before the start of the experiment, a total of 338479 microblog data points from 1306 users was initially crawled. After a preliminary manual cleaning and removal of some zombie users to ensure that a user’s microblog data was sufficient to meet the needs of gender identification, a weight of less than 10 users led to the microblog being removed. Finally, a total of 933 Sina Weibo users were left, of which 416 were male and 577 were female, which was a roughly balanced quantity.

It is necessary to determine a reasonable separation factor for the extraction and separation of the gender-specific thesauruses. In this paper, the separation factor is increased from 1.1 to 4.0 with a step size of 0.1, and 40 pairs of different gender-specific thesauruses are constructed. Based on these 40 pairs of different proprietary thesauruses with the 15 features mentioned in 3.2 to 3.4, 40 different experimental data were obtained. By sequentially validating the 40 data sets using the selected machine learning methods, a suitable separation factor can be obtained, which gives the best effect for gender identification.

### Feature validity verification

**Fig. 3:**
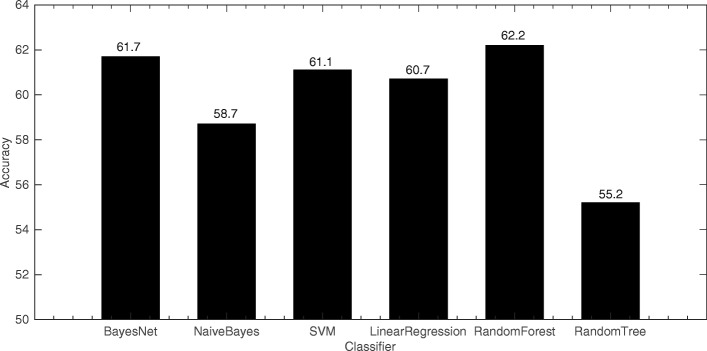
Traditional statistical features experiment histogram

Compared with the traditional statistics-based gender identification methods, this paper introduces an improved text implicitness feature and two features based on the construction of a gender-specific thesaurus. To validate the validity of the new features, six kinds of machine learning algorithms, such as the Random Forest classification algorithm were used to perform gender identification experiments (i.e., using features No.1 to No.13) based on traditional statistical features. The experiment results are shown in Fig. 3.

Taking the separation factor of 2.0 as an example, these six classification algorithms were reused to perform further experiments that added the improved text implicitness features and the two features based on constructing the gender-specific thesaurus. The experimental results are shown as follows.

**Fig. 4:**
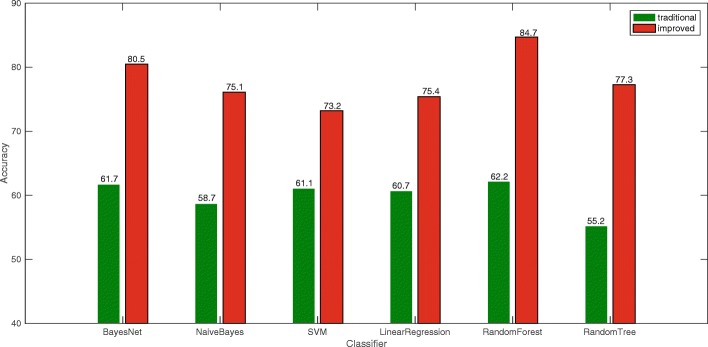
The total number of words in the gender-specific thesauruses comparison chart

In light of Fig. 4, it can be seen that the experimental effect has been greatly improved after the inclusion of the improved text implicitness feature and the two features based on the construction of the gender-specific thesauruses. This proves that the newly added features make sense for the effects of gender identification.

### Gender-specific thesaurus analysis

**Fig. 5:**
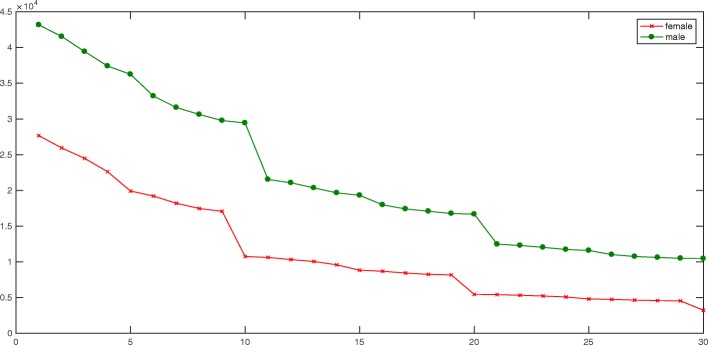
Comparison of classification results via a histogram

From Fig. 5, we constructed a set of 30 different gender-specific thesauruses through the process of constructing the gender lexicon proposed in “The extraction of gender-specific thesaurus” section. In this paper, we first extracted 45 thousand male-specific words and 27 thousand female-specific words from the nearly 330 thousand corpus (a total of 4.5 million words) to improve the gender representation of words. The gender difference in the number of words is due to the diversity of topics discussed by the users collected. Overall, the number of words in the male-specific thesaurus is greater than the number of the female-specific thesaurus. However, as continues to grow, the number of exclusive representations in the thesaurus continues to increase, and the number of terms in the gender-specific thesaurus declines. Furthermore, the gender representation of the remaining words in the thesaurus also rises.

Table 5 shows the part-of-speech statistics of the gender-specific thesaurus when *θ*=1.1, *θ*=2.0, *θ*=3.0 and *θ*=4.0. Table 5 also shows that the most obvious difference is in English words, then in nouns and verbs. With the ascent of *θ*, the three part-of-speech numbers have decreased but still occupy the top three spots of the gender-specific thesaurus. Note that the proportion of interjections in the thesaurus has always been low. When *θ*>3, the interjection does not exist in the male-specific thesaurus, but there is still a small fraction that exists in the female-specific thesaurus. This result shows that some interjections are basically only used by female users. When the amount of data in the basic corpus is large enough, we may be able to extract some special interjections and use them to improve the recognition accuracy of women.

**Table 5:** Gender-specific thesaurus-based feature

	*θ*=1.1		*θ*=2.0		*θ*=3.0		*θ*=4.0	
Sex_label	Male	Female	Male	Female	Male	Female	Male	Female
English_num	16316	10668	11370	4466	6646	2370	4309	1391
Noun_num	8475	5753	6333	2307	3555	1187	2229	724
Adj_num	1054	847	546	287	270	139	151	75
Prep_num	33	11	15	0	7	0	3	0
Pron_num	214	123	132	45	73	25	43	18
Verb_num	7625	4277	4705	1436	2633	639	1627	353
Adv_num	571	318	332	84	170	35	100	16
Int_num	8	5	3	3	1	2	0	2
Idiom_num	1565	1236	1068	457	535	217	283	128
Symbol_num	619	465	437	228	256	154	183	112
Others	6685	3962	4501	1438	2529	685	1534	405

### Classification algorithm comparison

In this paper, the classification algorithms of the Bayes Net, Naïve Bayes, SVM, Linear Regression (hereinafter abbreviated as LinearR), Random Tree and Random Forest are selected for machine verification. The SVM and the Linear Regression are common classification algorithms for dichotomous problem experiments, and the SVM has been proved to have excellent classification results in the research of gender identification. The Naive Bayes and Bayes Net are used in statistical classification problems as the classical classification algorithm. The Random Forest algorithm has the unique advantage of avoiding data overfitting and calculating feature contribution rankings.

The above six kinds of classification algorithms’ experimental results are shown below.

**Fig. 6:**
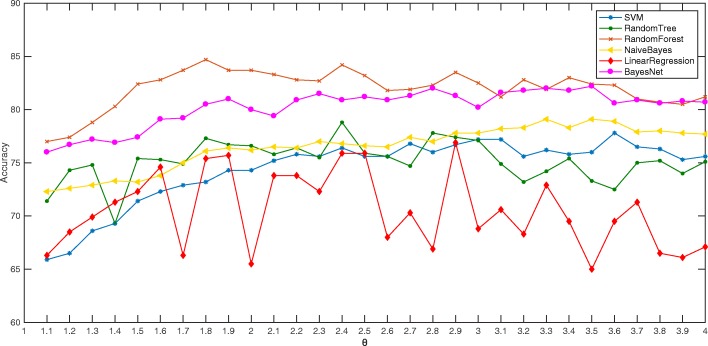
Traditional statistical features experiment histogram

From Fig. 6, the results of the experiments using the Random Forest algorithm are more stable than those of other classification algorithms, and the Bayes Net is the second most popular. Due to the multifeature problem, the performance of the LinearR is the most unsatisfactory and volatile. The SVM in this comparison did not show a significant advantage. However, with the accuracy of the gender-specific thesaurus division, the effect of the SVM was significantly improved. Overall, when the separation factor is taken as 1.8, the best classification result can be obtained using the Random Forest algorithm with an accuracy of 84.7%.

In the experiment, the gender-specific thesaurus has an influence on the experimental results, and the construction of the gender-specific thesaurus is influenced by *θ*. As the *θ* becomes larger, the representation of the words contained in the gender-specific thesaurus becomes more prominent. Therefore, in the initial stage where *θ* gradually increases from 1, the degree of preparation of classification becomes larger as *θ* becomes larger. When *θ* exceeds a certain value, the daily usage frequency of most words in the gender-specific thesaurus will decrease. When the words in the thesaurus are not words that people use frequently every day, the classifier will have difficulty capturing the gender features contained in the text, so the classification effect based on the thesaurus will begin to decrease. This is why in Fig. 6, the experimental results will rise first and then fall.

### Experimental comparison

**Fig. 7:**
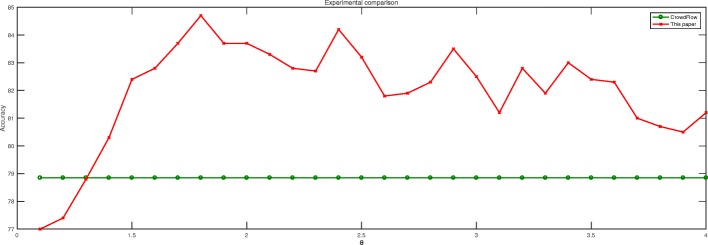
Experimental comparison histogram

This paper is compared with the CrowdFlower AI gender predictor, which is a gender prediction model developed by a large crowdsourcing company through extensive data training. From 2015, CrowdFlower AI mainly uses data to do AI direction research. CrowdFlower AI gender predictor is the product of related research. The code of this predictor is open source on GitHub. Crowdflower is a well-known platform, which is authoritative, so this paper selects the CrowdFlower AI gender predictor for comparison experiments, mainly using our dataset to reproduce CrowdFlower AI’s experiment. The experimental results are shown in Fig. 7.

The results of the comparative experiments are shown in Fig. 7. After experimentation, the experiment of CrowdFlower AI platform achieved the highest recognition accuracy of 78.84%. As can be seen from Fig. 7, the gender identification method based on the gender-specific thesauruses is better than the CrowdFlower AI result most of the time.The difference in experimentation is mainly due to the different languages used in the dataset. CrowdFlower AI is mainly used to train Twitter-based data, which is mainly in English. And the Chinese and English contexts still have relatively large differences, so the predictor does not perform very well on Chinese microblog data. In contrast, the method of this paper is more suitable for gender recognition work based on Chinese data.

### Feature contribution analysis

To further verify the validity of the attributes constructed based on the gender-specific thesaurus, the feature selection algorithm needs to be used to score the contribution of each feature. The Relief algorithm is used to calculate the feature contribution rates for dichotomous problems. The ReliefF algorithm is an improvement over the Relief algorithm, since it can make use of the nearest neighbor’s idea in the multidimensional space to get the contribution of the feature to the final classification result. Table 6 shows the contributions of the 15-dimensional features.

**Table 6:** Feature contribution ranking

Rank	Feature name	Importance
1	*FR*_*m*_	0.36
2	*UR*_*m*_	0.27
3	*Implict*	0.07
4	*Freq*_*emoj*_	0.04
5	*Sign*_*avg*_	0.04
6	*Noun*_*avg*_	0.03
7	*AVG*_*EWC*	0.03
8	*Idiom*_*avg*_	0.03
9	*Adv*_*avg*_	0.03
10	*Verb*_*avg*_	0.01
11	*Word*_*avg*_	0.02
12	*Adj*_*avg*_	0.02
13	*Pron*_*avg*_	0.02
14	*Prep*_*avg*_	0.01
15	*Interj*_*avg*_	0.01

The comparison shows that the two-dimensional attribute based on the gender-specific lexicon has the highest contribution rating to gender classification. The improved text implicitness feature is ranked in the third palce, which makes an excellent contribution to gender identification. The statistical characteristics of emoji expression also have a good contribution rate, because there is a significant difference in the frequency of emoji expression between men and women. Most of the traditional statistical features are located in lower positions, indicating that gender differences in terms of the part of speech are relatively small. After removing the statistical features, the recognition accuracy dropped to 81%, which was down 3.7% from its highest value at 84.7%, indicating that traditional statistical features still have some significance for gender identification work.

## Conclusions

This article addresses the issue of gender identification of Sina Weibo users and constructs a gender-specific thesaurus. On this basis, we construct two new features based on the frequency and universality of the use of words in the gender-specific thesaurus. After combining the traditional statistical features and improving the semantic complexity feature, the Random Forest algorithm achieved a highest classification efficiency of 84.7%. The experimental results show that there are differences in the choice of words between men and women, which are reflected in the differences in the gender-specific thesauruses. The new feature proposed in this paper based on the gender-specific thesaurus can effectively improve the classification accuracy.

At present, the research in this paper has certain limitations, because the gender of the users studied in this paper is limited to the premise of not considering gender camouflage. If gender camouflage is considered, the research perspective and consideration may not be the same as the current research. This is also an important direction for future research.

The gender-specific thesaurus proposed in this paper can reflect the difference between the two sexes, and the thesaurus can be changed according to the change of *θ*, so the thesaurus is not unique. In the current Chinese gender identification field, the thesaurus is rarely, this is a major breakthrough in this article, which also provides a lot of effective materials for scholars to study the differences between male and female users in the future. In this paper, we use the machine learning method to classify users. With higher classification accuracy, we will continue to explore other methods. For example, we will try to build a more efficient and accurate classification model based on the results of artificial neural networks to further improve the accuracy of gender classification and the classifier performance.
